# Halloysite Nanotubes Coated by Chitosan for the Controlled Release of Khellin

**DOI:** 10.3390/polym12081766

**Published:** 2020-08-07

**Authors:** Lorenzo Lisuzzo, Giuseppe Cavallaro, Stefana Milioto, Giuseppe Lazzara

**Affiliations:** 1 Dipartimento di Fisica e Chimica, Università degli Studi di Palermo, Viale delle Scienze, pad. 17, 90128 Palermo, Italy; lorenzo.lisuzzo@unipa.it (L.L.); stefana.milioto@unipa.it (S.M.); giuseppe.lazzara@unipa.it (G.L.); 2 Consorzio Interuniversitario Nazionale per la Scienza e Tecnologia dei Materiali, INSTM, Via G. Giusti, 9, I-50121 Firenze, Italy

**Keywords:** chitosan, halloysite nanotubes, khellin, release properties, thermogravimetry

## Abstract

In this work, we have developed a novel strategy to prepare hybrid nanostructures with controlled release properties towards khellin by exploiting the electrostatic interactions between chitosan and halloysite nanotubes (HNT). Firstly, khellin was loaded into the HNT lumen by the vacuum-assisted procedure. The drug confinement within the halloysite cavity has been proved by water contact angle experiments on the HNT/khellin tablets. Therefore, the loaded nanotubes were coated with chitosan as a consequence of the attractions between the cationic biopolymer and the halloysite outer surface, which is negatively charged in a wide pH range. The effect of the ionic strength of the aqueous medium on the coating efficiency of the clay nanotubes was investigated. The surface charge properties of HNT/khellin and chitosan/HNT/khellin nanomaterials were determined by ζ potential experiments, while their morphology was explored through Scanning Electron Microscopy (SEM). Water contact angle experiments were conducted to explore the influence of the chitosan coating on the hydrophilic/hydrophobic character of halloysite external surface. Thermogravimetry (TG) experiments were conducted to study the thermal behavior of the composite nanomaterials. The amounts of loaded khellin and coated chitosan in the hybrid nanostructures were estimated by a quantitative analysis of the TG curves. The release kinetics of khellin were studied in aqueous solvents at different pH conditions (acidic, neutral and basic) and the obtained data were analyzed by the Korsmeyer–Peppas model. The release properties were interpreted on the basis of the TG and ζ potential results. In conclusion, this study demonstrates that halloysite nanotubes wrapped by chitosan layers can be effective as drug delivery systems.

## 1. Introduction

In recent years, hybrid nanomaterials composed by biopolymers and inorganic nanoparticles have attracted growing interest within several fields, including biomedicine [1,2,3,4,5], pharmaceutics [6,7,8,9,10], food packaging [11,12,13,14,15], remediation [16,17] and cultural heritage [18,19,20]. As evidenced in a recent review [21], both ionic and non-ionic polysaccharides can be suitable polymers for the development of functional nanocomposites, with excellent performances in terms of thermal stability, barrier properties and mechanical behavior. Among the polysaccharides, chitosan was largely employed because of its chemical properties (such as hydrophobicity and pH sensitive features) as well as its antibacterial capacity [22]. It should be noted that the properties and the consequent applications of chitosan depend on both its molecular weight and the deacetylation degree [23]. Smart bionanocomposites were obtained by filling the chitosan matrix with both synthetic and natural nanoparticles. Among synthetic fillers, metal nanoparticles (Cu, Ag and Au) were deposited on chitosan supports [24]. The microwave heating technique was employed to fabricate chitosan/ZnO nanoaparticle hybrids with excellent removal capacity towards methylene blue [16], while the casting method was used to prepare chitosan/CuO nanoparticles’ composite films as H_2_S gas sensor membranes [25]. Multiwalled carbon nanotubes (MWCNT) were added to chitosan hydrogel in order to obtain composite scaffolds for sensing nitrofurantoin [2]. Concerning the natural fillers, nanoclays represent a green option to generate hybrids based on chitosan. Composite films with antibacterial properties were obtained by filling the chitosan/glycerol blend with several clay nanoparticles (bentonite, sepiolite and montmorillonite), which possess different morphological characteristics [26]. Variable contents of nano-hydroxyapatite were incorporated within the chitosan matrix to fabricate composite scaffolds with enhanced elasticity and flexibility [27]. An injectable viscous mucus was achieved by adding rectorite clay nanoparticles into chitosan [28]. Antibacterial and antioxidative films were prepared by mixing chitosan and kaolinite nanosheets [29]. Moreover, the chitosan/kaolinite nanocomposites exhibited better mechanical performances (in terms of tensile strength) with respect to those of the pristine biopolymer [29]. Recently, halloysite was largely investigated as a filler of chitosan in different phases, including aqueous suspensions [21,30], hydrogels [6,17] and solid films [1,31]. Composite scaffolds for tissue engineering purposes were obtained by filling chitosan with halloysite through the solution-mixing and freeze-drying methods [31]. The prepared chitosan/halloysite composite exhibited improved thermo-mechanical performances compared to those of pure chitosan [31]. Gel beads formed by chitosan and halloysite were revealed to be efficient removal agents towards dyes [17] and proper drug delivery systems for doxycycline [6]. Halloysite presents a hollow tubular shape with sizes dependent on its geological source [14]. Halloysite nanotubes (HNTs) possess an average length of 1 μm, while the inner and outer diameters range between 5–70 and 20–150 nm, respectively [14]. Interestingly, the pore volume of halloysite can be increased by acid treatment of its inner surface [32,33]. Due to their morphological characteristics, halloysite nanotubes are efficient as catalytic supports [34,35,36,37], adsorbents of pollutants [38,39] and nanocarriers for functional molecules with biological [40,41,42,43,44,45] and chemical activities [46,47,48,49]. In this regards, it should be highlighted that halloysite is suitable for biomedical and pharmaceutical applications because of its biocompatibility and low toxicity, which was observed to both unicellular and multicellular organisms [50,51,52,53,54]. Interestingly, the inside/outside surfaces of halloysite are oppositely charged within the pH interval between 2 and 8 [55]. This peculiarity was exploited for the targeted functionalization of halloysite nanotubes through ionic molecules, such as polyelectrolytes [30,56], proteins [57,58] and surfactants [43,59]. Structural investigations by electric birefringence and fluorescence correlation spectroscopy evidenced that the cationic chitosan wraps the clay nanotubes as a consequence of its selective adsorption onto the negative external surface of halloysite [56]. Here, we prepared a novel nanocarrier for khellin by exploiting the electrostatic attractions of chitosan with the halloysite outer surface. The chitosan coating of the nanoclay was designed to control the khellin release under variable pH conditions, which simulate pharmaceutical applications. The release properties of the chitosan-coated nanotubes were correlated to their morphology, surface charge characteristics and thermal behavior.

## 2. Materials and Methods

### 2.1. Materials

Halloysite nanotubes (HNT), chitosan (Mw = 50–190 kg mol^−1^, deacetylation degree 75–85%), khellin (Mw = 260.24 g mol^−1^), acetone, glacial acetic acid, sodium sodium hydroxide and hydrochloric acid (37% *v/v*) are Sigma Aldrich products (St. Louis, MO, USA). All products were used without any further purification.

### 2.2. Khellin Loading within Halloysite Cavity

Halloysite cavity was filled with khellin by using the procedure reported elsewhere for HNT/drug composites [6]. Firstly, we prepared a khellin saturated solution in acetone. Then, we added halloysite powder to the khellin solution in order to obtain a dispersion with HNT/khellin mass ratio equals to 1:1. The dispersion was ultrasonicated for 10 min and, subsequently, it was magnetically stirred for 24 h. Afterwards, the HNT/khellin suspension was kept under vacuum (*p* = 0.01 atm for 1 h) to favour the encapsulation of the drug within the halloysite cavity as a consequence of the Gibbs–Thomson effect [60]. The latter step was repeated three times in order to enhance the amount of khellin loaded into halloysite nanotubes. According to the literature [61], the dispersion was magnetically stirred for 10 min between two consecutive vacuum steps. Finally, the HNT/khellin composite was separated by centrifugation, which was conducted for 10 min at 7000 rpm. The solid material was rinsed with water three times to remove the unbound khellin, and then dried under vacuum. All steps were carried out at room temperature.

### 2.3. Chitosan Coating of Halloysite/Khellin Composite

The HNT/khellin composite was coated by chitosan, exploiting the electrostatic interactions occurring between the cationic biopolymer and the halloysite outer surface, which possesses a negative charge within a large pH interval (2–8) [55]. Firstly, chitosan was solubilized in 0.5 wt % acetic acid solution by magnetically stirring for 2 h. The chitosan concentration was set at 1 wt %. The chitosan solution was mixed with HNT aqueous dispersion (concentration equals 1 wt %). It should be noted that the HNT dispersion was prepared in two different media (water and 0.5 mol L^−1^ NaCl aqueous solution) in order to explore the effect of the ionic strength on the chitosan-coating efficiency. The obtained mixtures were ultrasonicated for 2 min and, subsequently, stirred for 30 min. Finally, the chitosan/HNT/khellin composites were separated by the aqueous solution through centrifugation (10 min at 7000 rpm). Similarly to the khellin loading into HNT cavity, all steps were performed at room temperature. 

### 2.4. Khellin Release Experiments

The kinetics of khellin release from HNT and chitosan-coated HNT nanocarriers were investigated by UV-VIS spectroscopy (Analytik, Jena, Germany) using the approach reported elsewhere [61]. Specifically, 100 mg of the composite (HNT/khellin or chitosan/HNT/khellin samples) was immersed in 30 mL of water (pH = 6), which represents the release medium. After a fixed time interval, 2 mL of aqueous medium was taken and investigated by UV-Vis spectroscopy to determine the khellin concentration. Meanwhile, 2 mL of fresh solution (water at pH = 6) was added to the release medium in order to maintain its volume at a constant. The release experiments were conducted up to 5 h. As V and V_0_ were equal to 2 and 30 mL, respectively, the corrected khellin concentrations in the release medium at different times were calculated as follows [61]

(1) $$C’n=\mathrm{Cn}+(V/V_{0})\cdot \sum_{i=0}^{n-1}\mathrm{Ci}$$

where C’n and Cn represent the corrected and the measured khellin concentrations, respectively. The khellin release data were determined by considering the C’n values.

### 2.5. Methods

#### 2.5.1. ζ Potential 

ζ-potential measurements were conducted by Zetasizer Nano-ZS (Malvern Instruments, London, UK) using disposable folded capillary cells. The experiments were conducted on 0.005 wt % aqueous dispersions of the investigated nanomaterials in isothermal conditions (T = 25 °C). Prior to the measurements, the suspensions were ultrasonicated for 5 min to avoid the aggregation of the nanoparticles. 

#### 2.5.2. Scanning Electron Microscopy (SEM)

SEM analyses in high vacuum (<6 × 10^−4^ Pa) were carried out through the ESEM FEI QUANTA 200F microscope (Hillsboro, OR, USA). The energy of the beam was set at 25 kV, while the working distance was fixed at 10 mm.

#### 2.5.3. Thermogravimetry 

Thermogravimetric experiments were performed by using a Q5000 IR apparatus (TA Instruments, New Castle, DE, USA). The measurements were conducted under inert atmosphere using nitrogen flows for the sample and the balance (25 and 10 cm^3^ min^−1^, respectively). The samples were heated (scanning rate of 20 °C min^−1^) from 25 to 800 °C. According to the literature [62,63], the temperature calibration was carried out by exploiting the Curie temperatures of specific standards (nickel, cobalt, and their alloys). Thermogravimetric measurements were repeated three times for all the investigated samples. The average values with the corresponding standard deviations for the thermogravimetric parameters are presented. 

#### 2.5.4. UV-VIS Spectroscopy 

UV-VIS spectra were registered by using the Specord S600 (Analytik, Jena, Germany) spectrophotometer within the wavelength range between 200 and 400 nm. To this purpose, quartz cuvettes were used. The khellin absorption peak at 250 nm was analyzed for the investigation of the release kinetics. 

#### 2.5.5. Water Contact Angle

Water contact angle experiments were conducted by using an optical contact angle apparatus (OCA 20, Data Physics Instruments, Filderstadt, Germany) equipped, with a video measuring system with a high-resolution CCD camera and a high-performance digitizing adapter. Data acquisition was conducted by SCA 20 software (Data Physics Instruments, Filderstadt, Germany). The initial contact angle (*θ_i_*) of water in air was detected through the sessile drop method by placing a water droplet of 10 ± 0.5 mL onto the surface of the sample tablets. The tests were performed at 25.0 ± 0.1 °C. Each sample was analyzed three times. The average values with the corresponding standard deviation for the initial water contact angle are reported. 

## 3. Results

### 3.1. Surface Charge and Morphological Characteristics

The surface charge of HNT/khellin and chitosan/HNT/khellin composite materials was investigated by ζ potential measurements. As shown in Table 1, the khellin loading into the HNT cavity did not significantly alter the ζ potential of halloyisite, avoiding any electrostatic interactions between the drug and the internal surface of the clay nanotubes. This finding agrees with the hydrophobic character of khellin, which is encapsulated within the HNT cavity as a consequence of the solvent confinement [60]. The khellin confinement into the halloysite cavity was confirmed by water contact angle experiments, which evidenced that the *θ_i_* values for HNT and HNT/khellin tablets are 30.5 ± 1.1° and 30.9 ± 1.2°, respectively. These results indicate that the khellin loading did not alter the hydrophilic character of the HNT outer surface, demonstrating that the hydrophobic drug is mostly confined to the halloysite cavity. 

As concerns the chitosan/HNT/khellin composites, we observed that the presence of NaCl strongly affects the *ζ* potential of the polymer coated nanotubes. In particular, an inversion of *ζ* potential (from negative to positive) was detected after the chitosan coating was conducted in NaCl aqueous solution. On this basis, we can assert that electrostatic attrations between cationic chitosan and the HNT outer surface (negatively charged) take place. As reported for alkyltrimetilammonium bromides/HNT hybrids [43], the effective adosrption of cationic molecules neutralizes the negative charges of the HNT shell, causing an inversion in the *ζ* potential. In contrast, the coating procedure in water generated a slight decrease in the HNT surface charge. These results highlighted that the chitosan coating efficiency is remarkably improved in aquoeus medium with higher ionic strength. The chitosan coating generated the hydrophobization of the HNT outer surface, as evidenced by the significant enhancements of the *θ*_i_ values (75 ± 2° and 80 ± 3° in water and NaCl solution, respectively) compared to that of the HNT/khellin composite (30.9 ± 1.2°). In this regard, Lvov et al. [64] evidenced that the addition of small amounts of electrolyte is used in the layer by layer technique because of the “rod-to-coil” transition of polyelectrolytes in solution. Additionally, it is reported that the increase in the ionic strength enhances the chitosan flexibility [65].

**Figure 1:**
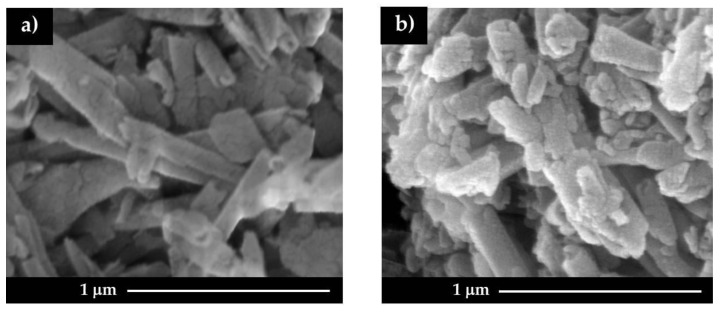
SEM images for HNT/khellin (**a**) and HNT/khellin coated with chitosan in NaCl solution (**b**).

SEM images (Figure 1) show that the hollow tubular morphology of halloysite was preserved in both HNT/khellin and chitosan/HNT/khellin hybrid prepared in NaCl solution. We observed that the nanotubes are glued to each other after their coating with chitosan.

### 3.2. Thermal Properties 

#### 3.2.1. HNT/Khellin 

**Figure 2:**
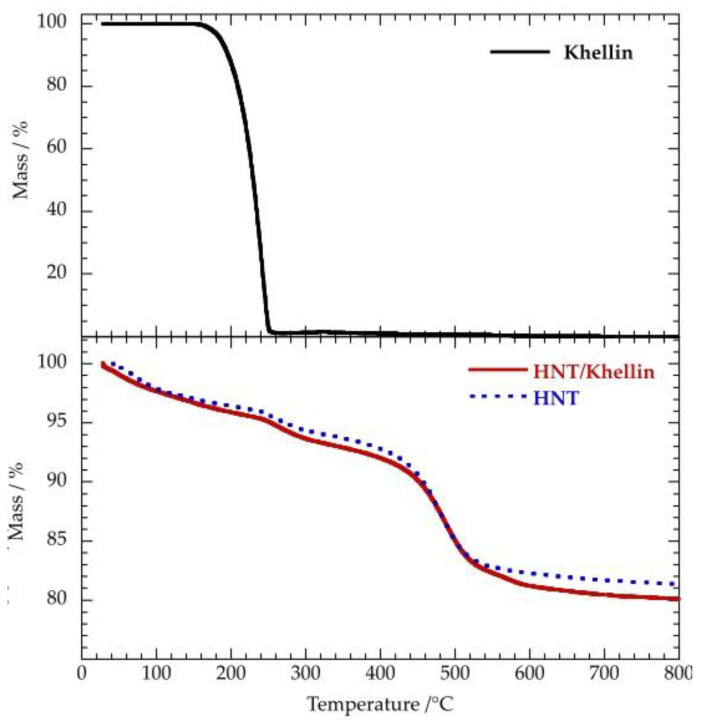
Thermogravimetric curves for Khellin, HNT and HNT/Khellin.

Figure 2 compares the thermogravimetric (TG) curve of HNT/khellin hybrid with those of the pristine components (HNT and khellin).

We observed that khellin totally decomposes in one single step occurring in the temperature range between 200 and 300 °C, while both HNT and HNT/khellin exhibit three different mass losses at 25–150, 200–300 and 450–550 °C. As reported elsewhere [66], the mass reduction from room temperature to 150 °C reflects the loss of the physically adsorbed water molecules. On the other hand, the mass change at 450–550 °C can be attributed to the expulsion of the interlayer water molecules that are present in the structure of halloysite mineral [66]. The mass change at 200–300 °C was enhanced in the loaded nanotubes, highlighting the successful khellin loading into the HNT cavity. According to the literature [60], we calculated the loading efficiency by considering the mass losses at 25–150 °C (ML_150_) and the residual masses at 800 °C (MR_800_) for the pure components and the composite material. Table 2 collects the ML_150_ and MR_800_ values of khellin, HNT and HNT/khellin samples. 

The degraded matters at 800 °C (MD_800_) for each sample was determined as

MD_800_ = 100 − (MR_800_ + ML_150_) (2)

Based on the rule of mixture [60], the MD_800_ of HNT/khellin composite (MD_800-KH_) can be expressed as

MD_800-HK_ = (C_H_ · MD_800-H_ + C_K_ · MD_800-K_)/100 (3)

where MD_800-K_ and MD_800-H_ are the degraded matters at 800 °C for khellin and HNT, respectively, whereas C_K_ and C_H_ represent the mass percentages of halloysite and khellin, respectively.

The combination of Equations (2) and (3) allowed us to estimate the khellin loading (1.03 ± 0.02 wt %) in the composite material on the basis of the thermogravimetric parameters presented in Table 2.

#### 3.2.2. Chitosan/HNT/Khellin

**Figure 3:**
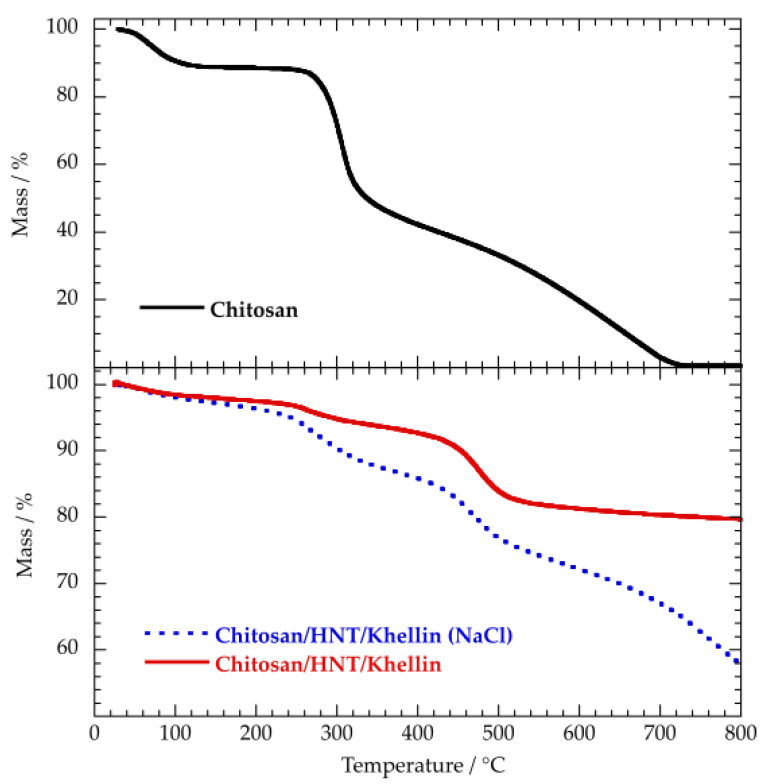
Thermogravimetric curves for chitosan and chitosan/HNT/khellin samples.

The thermal effects of the chitosan coating on the khellin loaded nanotubes were investigated by thermogravimetry. Within this, Figure 3 shows the TG curves for chitosan and chitosan/HNT/khellin composites. 

The TG curve of chitosan evidenced three mass losses that can be related to different processes. As reported in the previous paragraph, the mass reduction up to 150 °C reflects the moisture content of the material. The mass loss at 250–400 °C is due to the depolymerisation/decomposition of chitosan chains as a consequence of the deacetylation and cleavage of glycosidic bonds [67]. The last degradation step is caused by the decomposition of pyranose ring and residual carbon [68]. The corresponding mass loss was detected in the temperature interval between 400 and 700 °C. The thermogravimetric curves of the chitosan/HNT/khellin composites (Figure 3) evidenced that the presence of NaCl strongly affects the thermal behavior of the polymer coated nanotubes. As shown in Table 3, the MR_800_ value is much larger for the composite prepared with the electrolyte, highlighting its larger thermal stability.

Interestingly, the last degradation step of chitosan was clearly detected only for the composite prepared in the NaCl aqueous solution. This result could be quantitatively explained on the basis of ζ potential experiments, which highlighted that the chitosan-coating efficiency can be enhanced by increasing the ionic strength of the aqueous medium. In addition, we observed that the thermal decomposition of the pyranose ring and residual carbon is shifted to a larger temperature range, indicating that chitosan was thermally stabilized by its electrostatic interactions with the halloysite external surface. We calculated the chitosan coating efficiencies by using the rule of mixtures on the degraded masses at 800 °C, using the same approach employed for the determination of the khellin loading. Accordingly, the MD_800_ of chitosan/HNT/khellin (MD_800-CHK_) can be expressed by the following equation:

MD_800-CHK_ = (C_HK_ · MD_800-HK_ + C_C_ · MD_800-C_)/100 (4)

MD_800-C_ represents the degraded mass at 800 °C of chitosan, while C_C_ represents its mass percent in the coated nanotubes.

Based on Equations (2) and (4) and taking into account the thermogravimetric parameters (Table 3), we estimated that the biopolymer amount in the chitosan/HNT/khellin prepared in water is 2.38 ± 0.03 wt %. The coating efficiency was significantly improved by dispersing chitosan in NaCl aqueous solution and water, as evidenced by the biopolymer content (31.4 ± 0.3 wt %) of the coated nanotubes. These results could be related to the effect of the electrolyte on the chitosan chains’ flexibility.

### 3.3. Khellin Release Experiments 

**Figure 4:**
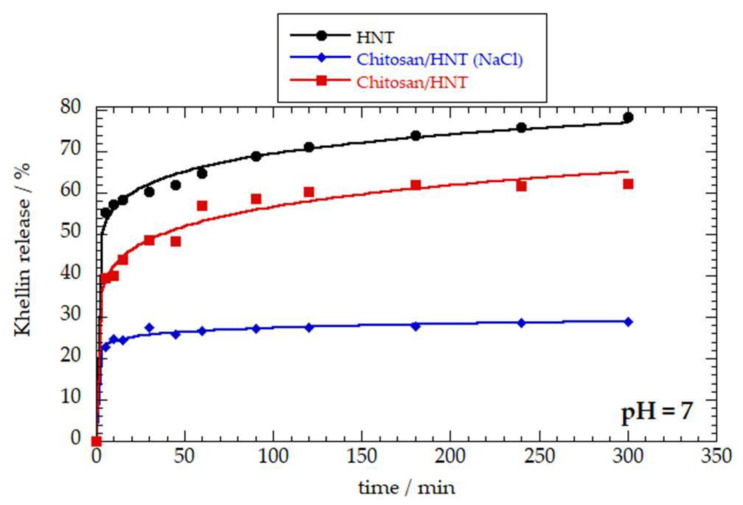
Khellin release as a function of time in water under neutral conditions (pH = 7). Solid lines represent the fitting according to the Korsmeyer–Peppas model (Equation (5)).

The effect of the chitosan coating on the khellin release from halloysite nanotubes was investigated at variable pH conditions. Figure 4 shows the khellin release kinetics under neutral conditions for HNT and HNT coated with chitosan. As a general result, the adsorption of chitosan onto the HNT outer surface slowed down the khellin release from the halloysite cavity. In this regard, the amount of khellin released after 6 h was 78% for uncoated nanotubes. This percentage was reduced in the chitosan/HNT nanocarriers (62 and 28% for the composites prepared in water and NaCl solution, respectively). These results are consistent with the ζ potential and TGA experiments, which evidenced that the presence of electrolyte improved the chitosan-coating efficiency of the nanotubes. Namely, the nanotubes coated in the NaCl solution are more effective in controlling the khellin release because of the larger amount of chitosan on the HNT shell. The khellin release profiles were analyzed using the Korsmeyer−Peppas model, which is expressed by the following equation

R% = k∙t*^n^* (5)

where R% is the drug percentage release at a certain time (t), while k and *n* are the kinetic constant and the release exponent, respectively. It should be noted that this equation is valid for R% ≤ 80%, as evidenced for drug release from polymer/HNT hybrids [43]. The obtained fitting parameters are presented in Table 4. Compared to the HNT-based carrier, the kinetic contact was reduced in the coated nanotubes, confirming the chitosan-retarding effect on the the drug release. As expected, this effect is stronger for the coated nanotubes obtained in the NaCl aqueous solution. In general, the release data reflect a Fickian diffusion mechanism being that the *n* values are lower than 0.43 for all the nanocarriers [43]. 

**Figure 5:**
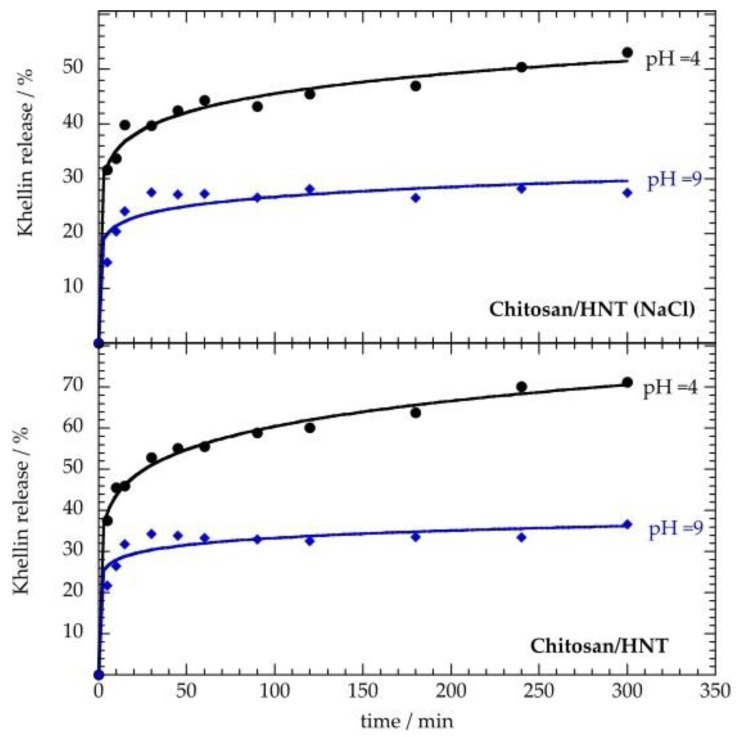
Khellin release as a function of time in water under acidic (pH = 4) and basic (pH = 9) conditions. Solid lines represent the fitting according to the Korsmeyer–Peppas model (Equation (5)).

We explored the influence of pH on the khellin release from chitosan/HNT hybrid carriers (Figure 5). As previously shown in Figure 4, the release data were fitted by using Equation (5). Table 5 shows that the chitosan retarding effect was reduced under acidic conditions, as evidenced by the lower k values with respect to those obtained at pH = 9. These results can be attributed to the enhanced chitosan solubility in water at pH ≤ 4 [69]. As observed at pH = 7, the khellin release is slower for the nanotubes coated in NaCl solution. This finding is valid for both basic and acidic conditions. Finally, the *n* values (<0.43) highlight that the release mechanism for all the investigated systems can be ascribed to the Fickian diffusion. 

## 4. Discussion

The application value of chitosan/HNT hybrid as nanocarrier for khellin was highlighted by the release experiments conducted at variable pH simulating the typical conditions along the human gastro-intestinal path, which moves from acidic (stomach) to basic (colon) conditions. Within this, it should be pointed out that khellin is largely used by oral administration. As a general result, the release kinetics were successfully analyzed by the Korsmeyer−Peppas model. The release mechanism (Fickian diffusion) was not affected by either pH or chitosan coating, with the release exponent (*n*) being ≤0.43 for all the kinetics (Table 4 and Table 5). We observed that the chitosan coated onto the nanotubes in NaCl solution is more effective in controlling the khellin release compared to those without NaCl. This observation is valid at pH = 7 (Figure 4) as well under acidic/basic conditions (Figure 5). These results could be related to the larger amount of coated chitosan onto the nanotubes in NaCl solution with respect to that in water as a consequence of the influence of the electrolyte on the flexibility of the polymeric chains. Specifically, the increase in the solvent ionic strength makes chitosan more flexible in agreement with the reduction in the repulsive interactions between the polymeric chains [65]. We can hypothesize that the chitosan coating layer onto HNT is thicker in NaCl solution and, consequently, the controlling efficiency on the khellin release is more effective. As shown in Figure 4 and Figure 5, the final drug release is lower than 100% for all the investigated systems in acidic, neutral and basic conditions. Similar observations were detected for the aspirin release from pristine and APTES modified halloysite, which exhibited final drug releases of 89 and 68%, respectively, at pH = 6.8 [70]. The final curcumin releases from thiahelicene-grafted HNT were ca. 10 and 3% at pH = 5 and 6.8, respectively [71]. Recent reviews [40,72] evidenced that halloysite nanotubes (both pristine and functionalized ones) are considered promising drug delivery systems, although the 100% release is not achieved. 

## 5. Conclusions

We demonstrated that the specific electrostatic attractions between halloysite outer surface and the cationic chitosan are effective to obtain composite tubular nanocarriers with controlled release properties towards khellin. The preparation of the chitosan/halloysite/khellin hybrid was conducted in aqueous medium on the basis of the following steps: (1) loading of khellin into the halloysite cavity; (2) chitosan coating of halloysite shell. The latter was carried out both in water and in NaCl aqueous medium to explore the effect of the ionic strength on the polymer coating efficiency. Scanning Electron Microscopy evidenced that the hollow tubular shape of halloysite was not altered by the khellin loading or the chitosan coating. The ζ potential of halloysite was not significantly affected by the encapsulated khellin (1.03 wt %), avoiding the presence of electrostatic interactions between the drug molecule and the nanotubes. In contrast, the surface charge of halloysite was influenced by the chitosan coating. Remarkably, an inversion in the halloysite ζ potential was detected by the chitosan wrapping conducted in NaCl solution, highlighting that the biopolymer/nanoclay interactions are favored by the electrolyte addition. According to these results, the coating efficiency was improved by ca. 10% in NaCl solution compared to that in water. Water contact angle experiments evidenced that the hydrophilic character of halloysite is not affected by khellin loading, confirming the drug’s confinement into the HNT cavity. On the other hand, the chitosan coating generated a significant hydrophobization of HNT/khellin composite as a consequence of the biopolymer adsorption onto the HNT shell. The presence of chitosan onto halloysite outer surface slowed down the khellin release. This effect is stronger for chitosan-coated nanotubes prepared in NaCl solution, as expected by ζ potential and thermogravimetric data. Release kinetics at variable pH evidenced that the chitosan retarding effect decreases under acidic conditions. Based on these considerations, we can conclude that the chitosan coating of halloysite nanotubes driven by electrostatic interactions can be considered a suitable strategy to obtain drug delivery systems with tunable properties. 

## Figures and Tables

**Table 1:** ζ potential for halloysite nanotubes (HNT), HNT/khellin and chitosan/HNT/khellin composite materials.

Material	ζ Potential/mV
HNT	−20.0 ± 0.6
HNT/khellin	−21.5 ± 0.3
Chitosan/HNT/khellin (NaCl)	+23.5 ± 0.8
Chitosan/HNT/khellin	−18.5 ± 0.4

**Table 2:** Thermogravimetric parameters of Khellin, HNT and HNT/Khellin composite.

Material	ML_150_/wt %	MR_800_/wt %	MD_800_/wt %
Khellin	0	0	100
HNT	2.93 ± 0.04	81.4 ± 1.2	15.7 ± 0.2
HNT/khellin	3.27± 0.04	80.2 ± 1.1	16.5± 0.2

**Table 3:** Thermogravimetric parameters of Chitosan and Chitosan/HNT/Khellin samples.

Material	ML_150_/wt %	MR_800_/wt %	MD_800_/wt %
Chitosan	11.3 ± 0.2	0	88.7 ± 1.5
Chitosan/HNT/Khellin (NaCl)	2.88 ± 0.03	57.9 ± 0.6	33.2 ± 0.3
Chitosan/HNT/Khellin	2.11 ± 0.03	79.6 ± 0.9	18.2 ± 0.3

**Table 4:** Fitting Parameters for khellin release at pH = 7.

Nanocarrier	k/min^−*n*^	*n*
HNT	45.46 ± 1.14	0.092 ± 0.006
Chitosan/HNT (NaCl)	21.6 ± 0.5	0.052 ± 0.006
Chitosan/HNT	31.7 ± 1.6	0.12 ± 0.01

**Table 5:** Fitting parameters for khellin release under acidic (pH = 4) and basic (pH = 9) conditions.

Nanocarrier	pH	k/min^−*n*^	*n*
Chitosan/HNT (NaCl)	4	27.2 ± 1.1	0.112 ± 0.009
Chitosan/HNT (NaCl)	9	17.2 ± 1.9	0.09 ± 0.02
Chitosan/HNT	4	31.6 ± 1.1	0.141 ± 0.007
Chitosan/HNT	9	23 ± 2	0.08 ± 0.02

## References

[1] Xie M., Huang K., Yang F., Wang R., Han L., Yu H., Ye Z., Wu F. (2020). Chitosan nanocomposite films based on halloysite nanotubes modification for potential biomedical applications. Int. J. Biol. Macromol..

[2] Velmurugan S., Palanisamy S., Yang T.C., Gochoo M., Chen S.-W. (2020). Ultrasonic assisted functionalization of MWCNT and synergistic electrocatalytic effect of nano-hydroxyapatite incorporated MWCNT-chitosan scaffolds for sensing of nitrofurantoin. Ultrason. Sonochem..

[3] Radwan-Pragłowska J., Janus Ł., Piątkowski M., Bogdał D., Matýsek D. (2020). Hybrid Bilayer PLA/Chitosan Nanofibrous Scaffolds Doped with ZnO, Fe_3_O_4_, and Au Nanoparticles with Bioactive Properties for Skin Tissue Engineering. Polymers.

[4] Nastyshyn S., Raczkowska J., Stetsyshyn Y., Orzechowska B., Bernasik A., Shymborska Y., Brzychczy-Włoch M., Gosiewski T., Lishchynskyi O., Ohar H. (2020). Non-cytotoxic, temperature-responsive and antibacterial POEGMA based nanocomposite coatings with silver nanoparticles. RSC Adv..

[5] Govindasamy K., Dahlan N.A., Janarthanan P., Goh K.L., Chai S.-P., Pasbakhsh P. (2020). Electrospun chitosan/polyethylene-oxide (PEO)/halloysites (HAL) membranes for bone regeneration applications. Appl. Clay Sci..

[6] Lisuzzo L., Cavallaro G., Parisi F., Milioto S., Fakhrullin R., Lazzara G. (2019). Core/Shell Gel Beads with Embedded Halloysite Nanotubes for Controlled Drug Release. Coatings.

[7] Koosha M., Hamedi S. (2019). Intelligent Chitosan/PVA nanocomposite films containing black carrot anthocyanin and bentonite nanoclays with improved mechanical, thermal and antibacterial properties. Prog. Org. Coat..

[8] Wu Y., Rashidpour A., Almajano M.-P., Metón I. (2020). Chitosan-Based Drug Delivery System: Applications in Fish Biotechnology. Polymers.

[9] Rebitski E.P., Souza G.P., Santana S.A.A., Pergher S.B.C., Alcântara A.C.S. (2019). Bionanocomposites based on cationic and anionic layered clays as controlled release devices of amoxicillin. Appl. Clay Sci..

[10] Rebitski E.P., Alcântara A.C.S., Darder M., Cansian R.L., Gómez-Hortigüela L., Pergher S.B.C. (2018). Functional Carboxymethylcellulose/Zein Bionanocomposite Films Based on Neomycin Supported on Sepiolite or Montmorillonite Clays. ACS Omega.

[11] García-Quiles L., Valdés A., Cuello Á.F., Jiménez A., Garrigós M.C., Castell P. (2019). Reducing off-Flavour in Commercially Available Polyhydroxyalkanoate Materials by Autooxidation through Compounding with Organoclays. Polymers.

[12] Gorrasi G. (2015). Dispersion of halloysite loaded with natural antimicrobials into pectins: Characterization and controlled release analysis. Carbohydr. Polym..

[13] Gorrasi G., Pantani R., Murariu M., Dubois P. (2014). PLA/Halloysite Nanocomposite Films: Water Vapor Barrier Properties and Specific Key Characteristics. Macromol. Mater. Eng..

[14] Cavallaro G., Chiappisi L., Pasbakhsh P., Gradzielski M., Lazzara G. (2018). A structural comparison of halloysite nanotubes of different origin by Small-Angle Neutron Scattering (SANS) and Electric Birefringence. Appl. Clay Sci..

[15] Makaremi M., De Silva R.T., Pasbakhsh P. (2015). Electrospun Nanofibrous Membranes of Polyacrylonitrile/Halloysite with Superior Water Filtration Ability. J. Phys. Chem. C.

[16] Mostafa M.H., ElSawy M.A., Darwish M.S.A., Hussein L.I., Abdaleem A.H. (2020). Microwave-Assisted preparation of Chitosan/ZnO nanocomposite and its application in dye removal. Mater. Chem. Phys..

[17] Peng Q., Liu M., Zheng J., Zhou C. (2015). Adsorption of dyes in aqueous solutions by chitosan–halloysite nanotubes composite hydrogel beads. Microporous Mesoporous Mater..

[18] Cavallaro G., Milioto S., Nigamatzyanova L., Akhatova F., Fakhrullin R., Lazzara G. (2019). Pickering Emulsion Gels Based on Halloysite Nanotubes and Ionic Biopolymers: Properties and Cleaning Action on Marble Surface. ACS Appl. Nano Mater..

[19] Cavallaro G., Milioto S., Lazzara G. (2020). Halloysite Nanotubes: Interfacial Properties and Applications in Cultural Heritage. Langmuir.

[20] Cavallaro G., Lazzara G., Milioto S., Parisi F., Ruisi F. (2017). Nanocomposites based on esterified colophony and halloysite clay nanotubes as consolidants for waterlogged archaeological woods. Cellulose.

[21] Bertolino V., Cavallaro G., Milioto S., Lazzara G. (2020). Polysaccharides/Halloysite nanotubes for smart bionanocomposite materials. Carbohydr. Polym..

[22] Li J., Wu Y., Zhao L. (2016). Antibacterial activity and mechanism of chitosan with ultra high molecular weight. Carbohydr. Polym..

[23] Rinaudo M. (2006). Chitin and chitosan: Properties and applications. Prog. Polym. Sci..

[24] Rubina M., Shulenina A., Svetogorov R., Vasilkov A. (2020). Metal-Chitosan Nanocomposites: A Perspective Way to Preparation, Morphology, and Structural Studies. Macromol. Symp..

[25] Ali F.I., Mahmoud S.T., Awwad F., Greish Y.E., Abu-Hani A.F. (2020). Low power consumption and fast response H2S gas sensor based on a chitosan-CuO hybrid nanocomposite thin film. Carbohydr. Polym..

[26] Benucci I., Liburdi K., Cacciotti I., Lombardelli C., Zappino M., Nanni F., Esti M. (2018). Chitosan/clay nanocomposite films as supports for enzyme immobilization: An innovative green approach for winemaking applications. Food Hydrocoll..

[27] Ying R., Wang H., Sun R., Chen K. (2020). Preparation and properties of a highly dispersed nano-hydroxyapatite colloid used as a reinforcing filler for chitosan. Mater. Sci. Eng. C.

[28] Li X., Li Y.-C., Chen M., Shi Q., Sun R., Wang X. (2018). Chitosan/rectorite nanocomposite with injectable functionality for skin hemostasis. J. Mater. Chem. B.

[29] Neji A.B., Jridi M., Kchaou H., Nasri M., Sahnoun R.D. (2020). Preparation, characterization, mechanical and barrier properties investigation of chitosan-kaolinite nanocomposite. Polym. Test..

[30] Bertolino V., Cavallaro G., Lazzara G., Milioto S., Parisi F. (2017). Biopolymer-Targeted Adsorption onto Halloysite Nanotubes in Aqueous Media. Langmuir.

[31] Liu M., Wu C., Jiao Y., Xiong S., Zhou C. (2013). Chitosan–halloysite nanotubes nanocomposite scaffolds for tissue engineering. J. Mater. Chem. B.

[32] Lim S., Park S., Sohn D. (2020). Modification of halloysite nanotubes for enhancement of gas-adsorption capacity. Clays Clay Miner..

[33] Joo Y., Sim J.H., Jeon Y., Lee S.U., Sohn D. (2013). Opening and blocking the inner-pores of halloysite. Chem. Commun..

[34] Sadjadi S., Heravi M.M., Kazemi S.S. (2018). Ionic liquid decorated chitosan hybridized with clay: A novel support for immobilizing Pd nanoparticles. Carbohydr. Polym..

[35] Liu Y., Guan H., Zhang J., Zhao Y., Yang J., Zhang B. (2018). Polydopamine-coated halloysite nanotubes supported AgPd nanoalloy: An efficient catalyst for hydrolysis of ammonia borane. Int. J. Hydrogen Energy.

[36] Liu Y., Zhang J., Guan H., Zhao Y., Yang J.-H., Zhang B. (2018). Preparation of bimetallic Cu-Co nanocatalysts on poly (diallyldimethylammonium chloride) functionalized halloysite nanotubes for hydrolytic dehydrogenation of ammonia borane. Appl. Surf. Sci..

[37] Feng Y., Zhou X., Yang J.-H., Gao X., Yin L., Zhao Y., Zhang B. (2020). Encapsulation of Ammonia Borane in Pd/Halloysite Nanotubes for Efficient Thermal Dehydrogenation. ACS Sustain. Chem. Eng..

[38] Deng L., Yuan P., Liu D., Du P., Zhou J., Wei Y., Song Y., Liu Y. (2019). Effects of calcination and acid treatment on improving benzene adsorption performance of halloysite. Appl. Clay Sci..

[39] Song Y., Yuan P., Du P., Deng L., Wei Y., Liu D., Zhong X., Zhou J. (2020). A novel halloysite–CeOx nanohybrid for efficient arsenic removal. Appl. Clay Sci..

[40] Lvov Y., Wang W., Zhang L., Fakhrullin R. (2015). Halloysite Clay Nanotubes for Loading and Sustained Release of Functional Compounds. Adv. Mater..

[41] Dzamukova M.R., Naumenko E.A., Lvov Y.M., Fakhrullin R. (2015). Enzyme-activated intracellular drug delivery with tubule clay nanoformulation. Sci. Rep..

[42] Vergaro V., Lvov Y.M., Leporatti S. (2012). Halloysite Clay Nanotubes for Resveratrol Delivery to Cancer Cells. Macromol. Biosci..

[43] Cavallaro G., Lazzara G., Milioto S., Parisi F., Evtugyn V., Rozhina E., Fakhrullin R. (2018). Nanohydrogel Formation within the Halloysite Lumen for Triggered and Sustained Release. ACS Appl. Mater. Interfaces.

[44] Fizir M., Dramou P., Dahiru N.S., Ruya W., Huang T., He H. (2018). Halloysite nanotubes in analytical sciences and in drug delivery: A review. Microchim. Acta.

[45] Dramou P., Fizir M., Taleb A., Itatahine A., Dahiru N.S., Mehdi Y.A., Wei L., Zhang J., He H. (2018). Folic acid-conjugated chitosan oligosaccharide-magnetic halloysite nanotubes as a delivery system for camptothecin. Carbohydr. Polym..

[46] Liu F., Bai L., Zhang H., Song H., Hu L., Wu Y., Ba X. (2017). Smart H2O2-Responsive Drug Delivery System Made by Halloysite Nanotubes and Carbohydrate Polymers. ACS Appl. Mater. Interfaces.

[47] Cheng C., Gao Y., Song W., Zhao Q., Zhang H., Zhang H. (2020). Halloysite nanotube-based H2O2-responsive drug delivery system with a turn on effect on fluorescence for real-time monitoring. Chem. Eng. J..

[48] Cavallaro G., Milioto S., Parisi F., Lazzara G. (2018). Halloysite Nanotubes Loaded with Calcium Hydroxide: Alkaline Fillers for the Deacidification of Waterlogged Archeological Woods. ACS Appl. Mater. Interfaces.

[49] Joshi A., Abdullayev E., Vasiliev A., Volkova O., Lvov Y. (2013). Interfacial Modification of Clay Nanotubes for the Sustained Release of Corrosion Inhibitors. Langmuir.

[50] Zhao X., Wan Q., Fu X., Meng X., Ou X., Zhong R., Zhou Q., Liu M. (2019). Toxicity Evaluation of One-Dimensional Nanoparticles Using Caenorhabditis elegans: A Comparative Study of Halloysite Nanotubes and Chitin Nanocrystals. ACS Sustain. Chem. Eng..

[51] Fakhrullina G.I., Akhatova F.S., Lvov Y.M., Fakhrullin R. (2015). Toxicity of halloysite clay nanotubes in vivo: A Caenorhabditis elegans study. Environ. Sci. Nano.

[52] Kryuchkova M., Danilushkina A., Lvov Y., Fakhrullin R. (2016). Evaluation of toxicity of nanoclays and graphene oxide in vivo: A Paramecium caudatum study. Environ. Sci. Nano.

[53] Long Z., Wu Y.-P., Gao H.-Y., Zhang J., Ou X., He R.-R., Liu M. (2018). In vitro and in vivo toxicity evaluation of halloysite nanotubes. J. Mater. Chem. B.

[54] Wang X., Gong J., Rong R., Gui Z., Hu T., Xu X. (2018). Halloysite Nanotubes-Induced Al Accumulation and Fibrotic Response in Lung of Mice after 30-Day Repeated Oral Administration. J. Agric. Food Chem..

[55] Vergaro V., Abdullayev E., Lvov Y.M., Zeitoun A., Cingolani R., Rinaldi R., Leporatti S., Rinaldi R. (2010). Cytocompatibility and Uptake of Halloysite Clay Nanotubes. Biomacromolecules.

[56] Cavallaro G., Chiappisi L., Gradzielski M., Lazzara G. (2020). Effect of the supramolecular interactions on the nanostructure of halloysite/biopolymer hybrids: A comprehensive study by SANS, fluorescence correlation spectroscopy and electric birefringence. Phys. Chem. Chem. Phys..

[57] Tully J., Yendluri R., Lvov Y. (2016). Halloysite Clay Nanotubes for Enzyme Immobilization. Biomacromolecules.

[58] Cavallaro G., Milioto S., Konnova S., Fakhrullina G., Akhatova F., Lazzara G., Fakhrullin R., Lvov Y. (2020). Halloysite/Keratin Nanocomposite for Human Hair Photoprotection Coating. ACS Appl. Mater. Interfaces.

[59] Cavallaro G., Grillo I., Gradzielski M., Lazzara G. (2016). Structure of Hybrid Materials Based on Halloysite Nanotubes Filled with Anionic Surfactants. J. Phys. Chem. C.

[60] Lisuzzo L., Cavallaro G., Pasbakhsh P., Milioto S., Lazzara G. (2019). Why does vacuum drive to the loading of halloysite nanotubes? The key role of water confinement. J. Colloid Interface Sci..

[61] Lisuzzo L., Cavallaro G., Milioto S., Lazzara G. (2019). Layered composite based on halloysite and natural polymers: A carrier for the pH controlled release of drugs. New J. Chem..

[62] Blanco I., Abate L., Bottino F.A., Bottino P. (2014). Thermal behaviour of a series of novel aliphatic bridged polyhedral oligomeric silsesquioxanes (POSSs)/polystyrene (PS) nanocomposites: The influence of the bridge length on the resistance to thermal degradation. Polym. Degrad. Stab..

[63] Abate L., Bottino F.A., Cicala G., Chiacchio M.A., Ognibene G., Blanco I. (2019). Polystyrene Nanocomposites Reinforced with Novel Dumbbell-Shaped Phenyl-POSSs: Synthesis and Thermal Characterization. Polymer.

[64] Lvov Y., Decher G., Moehwald H. (1993). Assembly, structural characterization, and thermal behavior of layer-by-layer deposited ultrathin films of poly (vinyl sulfate) and poly (allylamine). Langmuir.

[65] Morariu S., Brunchi C.-E., Bercea M. (2012). The Behavior of Chitosan in Solvents with Different Ionic Strengths. Ind. Eng. Chem. Res..

[66] Duce C., Ciprioti S.V., Ghezzi L., Ierardi V., Tiné M.R. (2015). Thermal behavior study of pristine and modified halloysite nanotubes. J. Therm. Anal. Calorim..

[67] Ziegler-Borowska M., Chełminiak D., Kaczmarek H. (2015). Thermal stability of magnetic nanoparticles coated by blends of modified chitosan and poly(quaternary ammonium) salt. J. Therm. Anal. Calorim..

[68] Corazzari I., Nisticò R., Turci F., Faga M.G., Franzoso F., Tabasso S., Magnacca G. (2015). Advanced physico-chemical characterization of chitosan by means of TGA coupled on-line with FTIR and GCMS: Thermal degradation and water adsorption capacity. Polym. Degrad. Stab..

[69] Fan M., Hu Q., Shen K. (2009). Preparation and structure of chitosan soluble in wide pH range. Carbohydr. Polym..

[70] Lun H., Ouyang J., Yang H. (2014). Natural halloysite nanotubes modified as an aspirin carrier. RSC Adv..

[71] Taroni T., Cauteruccio S., Vago R., Franchi S., Barbero N., Licandro E., Ardizzone S., Meroni D. (2020). Thiahelicene-grafted halloysite nanotubes: Characterization, biological studies and pH triggered release. Appl. Surf. Sci..

[72] Leporatti S. (2017). Halloysite clay nanotubes as nano-bazookas for drug delivery. Polym. Int..

